# Peripheral blood cytokine profiles predict the severity of SARS-CoV-2 infection: an EPIC^3^ study analysis

**DOI:** 10.1186/s12879-025-10914-6

**Published:** 2025-05-08

**Authors:** Xumin Li, Vivek Pakanati, Cindy Liu, Tracy Wang, Daniel Morelli, Anna Korpak, Aaron Baraff, Stuart N. Isaacs, Amy Vittor, Kyong-Mi Chang, Elizabeth Le, Nicholas L. Smith, Jennifer S. Lee, Jennifer M. Ross, Javeed A. Shah, for the EPIC3 Investigators, Mihaela Aslan, Kelly Cho, J. Michael Gaziano, Mark Holodniy, Christine M. Hunt, Anna M. Korpak, Dawn T. Provenzale, Christina Williams, Mary-Claire Roghmann, Karen KC Coffey, Leslie Les Katzel, Michelle Newman, Gwen L. Robinson, Eric Garshick, Emily Wan, Emma Busenkell, Selena Chom, Christina Collins, Colleen Hynes, Demerise Johnston, Erin McHugh, Peter Rivoira, Olivia Sterns, John Jack Sweeney, Caroline Truland, Makaila Wall, Cathy Zhang, Federico Perez, Robin L.P. Jump, Robert Bonomo, David Canaday, Margaret Tiktin, Sara Abdelrahim, Taissa A. Bej, Janet Briggs, Elizabeth Delancey-Niksa, Oteshia Hicks, Corinne Kowal, Alexandria Alex Nguyen, Lisa Padro, Roger Bedimo, Rohit Manaktala, Erik Guajardo, Antoinette Hamilton, Lisa Jones, Marcia Keller-Ray, Angela Dela Llana, Jacob Mathew, Jennifer Jen McClure, Erick Meermans, Erin Messick, Dindi Moore-Matthews, Van Nguyen, Abeer Zein, Lindsay Nicholson, Mary Bessesen, Rosa Cunningham, Teresa Derian, Theresa Dunn, Camila Hanson, Kelsey Moore, Kimberly Owens, Cameron Rogowski, Janel Vigil, Anna Wyrwa, Micah McClain, Ephraim Tsalik, Christopher Woods, James Everhart, Christopher Hostler, Maria Joyce, Jack Anderson, Marline Marlena Brown, Lynette Gehlhausen, Amanda Hittinger, Sara Hoffman, Tyffany Evans Locklear, Maria Miggs, Deborah Murray, Bradly Brad Nicholson, Ashlyn Press, Jaspreet Reen, Delisa Robinson, Gary Wang, Asmita Gupte, Alaina Ritter, Leslie Brown, Tempa Curry, Laura Dixon, Jennifer Gollwitzer, Rebecca Kokot, Debra Robertson

**Affiliations:** 1 VA Puget Sound Health Care System, Seattle, WA USA; 2 Division of Allergy and Infectious Diseases, University of Washington, Seattle, WA USA; 3 Corporal Michael J. Crescenz VA Medical Center, Philadelphia, PA USA; 4 Perelman School of Medicine, University of Pennsylvania, Philadelphia, PA USA; 5 VA Palo Alto Health Care System, Palo Alto, CA USA; 6 Department of Epidemiology, University of Washington, Seattle, WA USA; 7 Division of Endocrinology, Gerontology, and Metabolism, Stanford University, Palo Alto, CA USA; 8 North Florida/South Georgia Veterans Health System, Gainesville, FL USA; 9 Division of Infectious Disease and Global Medicine, University of Florida, Gainesville, FL USA

**Keywords:** COVID-19, SARS-CoV-2, Cytokines, LASSO regression, Random forest, XGBoost, Veterans, Predictive biomarkers, Illness severity

## Abstract

**Background:**

Predicting which patients will develop severe COVID-19 complications could improve clinical care. Peripheral blood cytokine profiles may predict the severity of SARS-CoV-2 infection, but none have been identified in US Veterans.

**Methods:**

We analyzed peripheral blood cytokine profiles from 202 participants in the EPIC^3^ study, a prospective observational cohort of US Veterans tested for SARS-CoV-2 across 15 VA medical centers. Illness severity was assessed based on the highest level documented during the first 60 days after recruitment. We correlated cytokine levels with illness severity using LASSO logistic regression, random forest, and XGBoost models on a 70% training set and calculated the AUC on a 30% test set.

**Results:**

LASSO regression identified 6 cytokines as predictors of SARS-CoV-2 severity with 77.3% AUC in the test set. Random forest and XGBoost models achieved an AUC of 80.4% and 80.7% in the test set, respectively. All models assigned a feature importance to each cytokine, with IP-10, MCP-1, and HGF consistently identified as key markers.

**Conclusions:**

Cytokine profiles are predictive of SARS-CoV-2 severity in US Veterans and may guide tailored interventions for improved patient management.

**Supplementary Information:**

The online version contains supplementary material available at 10.1186/s12879-025-10914-6.

## Introduction

COVID-19, caused by severe acute respiratory syndrome coronavirus 2 (SARS-CoV-2), is an infectious disease that has achieved global reach. The COVID-19 pandemic has had a significant impact on the United States, resulting in over 1.1 million fatalities across the country [1]. Within the Veterans Health Administration (VHA), which is among the largest integrated health care systems in the U.S., over 870,000 infections with SARS-CoV-2 and approximately 24,000 deaths were attributed to COVID-19 [2–4].

The clinical manifestations of COVID-19 are diverse. Although most patients experience symptoms that range from mild to moderate, approximately 15% of patients develop severe pneumonia, and of these, around 5% require admission to the intensive care unit (ICU) due to acute respiratory distress syndrome (ARDS), septic shock, or multiple organ dysfunction, often resulting in high fatality rates [5]. Two significant prognostic challenges are the insufficient identification of key cytokines associated with lethal outcomes and the difficulty in predicting which patients are at increased risk of developing severe illness and death [6].

Individuals suffering from severe SARS-CoV-2 infection often develop cytokine storm, which is a prominent feature that is linked with poor clinical outcomes and multiple organ dysfunction syndrome [7]. Recent research has identified cytokines such as MCP-3, IP-10, and IL-6 as reliable indicators for the advancement of COVID-19 [6]. Although current hospital diagnostics and variables like comorbidities and age help assess COVID-19 severity, they have limited ability to capture immune response heterogeneity, which is a key driver of disease progression. Severe SARS-CoV-2 infection marked by hyperinflammation and immune exhaustion can precede clinical deterioration, such as hypoxemia and organ failure. Cytokine profiling enables earlier risk detection and may allow for timely intervention. Moreover, unlike static risk factors, cytokine-based models can offer more personalized risk assessment and help guide targeted treatments. Identifying cytokine biomarkers for severe SARS-CoV-2 infection may enhance clinical decision-making and patient care by enabling early identification and tailored intervention for those at greatest risk [8].

The EPIC^3^ (Epidemiology, Immunology, and Clinical Characteristics of COVID-19) study, conducted within the Veterans Health Administration (VHA), is dedicated to detailing the epidemiological patterns and the natural progression of SARS-CoV-2 among the Veteran population. It further seeks to evaluate the relationship between host and viral elements and the intensity of the infection, as well as the emergence of immunity over time. In this manuscript, we present findings concerning the highest level of illness severity within the initial 60 days after recruitment. Participating Veterans contributed data by completing questionnaires, either conducted as interviews or filled out by the Veterans themselves, and by providing biospecimens; clinical data assessment was conducted accessing comprehensive VHA Electronic Health Records (EHR) [9]. Utilizing a subset of the broader EPIC^3^ study participants and leveraging the combination of the questionnaire, EHR, and biospecimen data, we identified a host cytokine profile that independently predicted SARS-CoV-2 infection severities.

## Methods

### Registration

The EPIC^3^ study is registered on ClinicalTrials.gov (NCT number: NCT05764083). More details regarding the registration can be found at https://clinicaltrials.gov/study/NCT05764083#more-information.

### Study design

EPIC^3^ is a prospective, observational cohort study. From July 2020 to September 2022, it enrolled Veteran inpatient and outpatient participants across 15 Veterans Affairs medical facilities. The EPIC^3^ data, including questionnaires and biospecimens, were systematically gathered at baseline (day 0) and then, when possible, on days 3, 7, 14, 21, and 28, followed by the 3rd, 6th, 12th, 18th, and 24th months post-enrollment. This analysis uses risk prediction modeling to identify cytokine biomarkers for illness severity among participants who tested positive for COVID-19.

### Study population

The inclusion criteria for the study stipulate that participants must be aged 18 or older, classified as an inpatient or outpatient at one of the participating Veterans Affairs medical centers from June 2020 to September 2022, and have undergone a SARS-CoV-2 RT-PCR test within three weeks prior to recruitment. From the pool of invited Veterans, 60% agreed to enroll, and 21% of these participants had research blood drawn to undergo a comprehensive panel of 45 cytokines assessed using the Luminex platform. Participants or their authorized representatives provided informed consent. The study was reviewed and approved by the VA Central IRB. The criteria for our analysis were not based on a target number of samples, rather the sample size was limited by the number of samples available in the biorepository that fit the pre-defined criteria. Thus, our study sample included those with a positive SARS-CoV-2 RT-PCR test result at enrollment and baseline measurements of 45 cytokines.

### Exposures

Participants with positive test outcomes of SARS-CoV-2 RT-PCR tests at recruitment were chosen for this analysis. Their classification as either inpatients or outpatients was established at the baseline. Demographic data, including age, sex, and race/ethnicity, were gleaned from the initial questionnaires or EHR. We calculated participants’ Charlson comorbidity index (CCI), a composite measure of medical comorbidities, using the method detailed by Quan and colleagues, with data from the EHR from the 2 years before enrollment [10].

A comprehensive panel of 45 cytokines was assessed using the Luminex platform (Vendor: Luminex Corporation) at PHRL. These cytokines included BDNF, EGF, Eotaxin, FGF-2, GM-CSF, GRO-α, HGF, IFN-α, IFN-γ, IL-1α, IL-1β, IL-1RA, IL-2, IL-4, IL-5, IL-6, IL-7, IL-8, IL-9, IL-10, IL-12(p70), IL-13, IL-15, IL-17A, IL-18, IL-21, IL-22, IL-23, IL-27, IL-31, IP-10, LIF, MCP-1, MIP-1-α, MIP-1-β, NGF-β, PDGF-BB, PlGF-1, RANTES, SCF, SDF-1-α, TNF-α, TNF-β, VEGF-A, and VEGF-D.

### Outcome

One outcome assessed for all study participants was the degree of illness severity. This metric was determined by the highest level of severity a participant experienced within the first 60 days following their entry into the study. Severity was measured using the Veterans Affairs Severity Index for COVID-19 (VASIC), which is an exclusive 4-tier scale (mild, moderate, severe, or death) derived from the World Health Organization’s COVID-19 severity scale. This scale was applied to EHR data, and its accuracy was confirmed through medical record review [11]. Mild severity encompassed participants who were in the hospital for 24 h or less; moderate severity was for those hospitalized for more than 24 h and included cases requiring low-flow oxygen therapy; severe cases were those needing high-flow oxygen, intubation, mechanical ventilation, extracorporeal membrane oxygenation, vasopressors, or initiation of renal dialysis within 60 days post-diagnosis; and any deaths occurring within 60 days, irrespective of cause, were classified under the death category.

In this analysis, the participants were stratified based on their SARS-CoV-2 infection severity into two categories: mild/moderate and severe/death. This approach addresses the limited sample size and simplifies the task of differentiating levels of COVID-19 severity. Moreover, combining'severe'and'death'categories addresses the issue of class imbalance due to the rarity of death events, enhancing the models’ ability to identify patterns predicting severe outcomes.

### Statistical analyses

The count and percentage of the participants’ baseline characteristics in each age, CCI, race/ethnicity, cohort (inpatient vs. outpatient), and sex categories were calculated, with each category further stratified by the severity of SARS-CoV-2 infection. The age groups were defined as: < 30, 30–39, 40–49, 50–59, 60–69, 70–79, and > = 80 years. In this study, participants were categorized into four CCI groups: 0, 1–2, 3–4, and 5+. The race and ethnicity of the participants were combined into a single race/ethnicity variable, that included the categories: Hispanic, Non-Hispanic Black, Non-Hispanic White, and Other. The cohort category identified a participant as either inpatient or outpatient, and the sex of participants was classified as female or male.

We also compared the baseline cytokine responses between the mild/moderate and severe/death groups. The results were presented as median and interquartile range (IQR). We calculated the percentage of out-of-detection-limit values for each cytokine. We divided participants into a 70% training set and a 30% test set using randomization stratified by the severity of their SARS-CoV-2 infection. We assessed whether the training and test sets were comparable in terms of the severity of SARS-CoV-2 infection and baseline characteristics. Then we conducted univariate analysis in the training set to examine the ability of each cytokine response to differentiate between mild/moderate vs severe/death cases by using the Receiver Operating Characteristic Curve (ROC) and the area under the ROC curve (AUC). We also calculated the sensitivity, specificity, Youden index, and best cut-off value for each model. After the univariate analysis, we chose the cytokines with AUC’s 95% confidence interval (CI) lower boundary exceeding 0.5 (random guess) to be included in the risk prediction models. Additionally, we evaluated the ability of CCI alone to distinguish between mild/moderate and severe/death cases using ROC and AUC analysis.

We leveraged the Pearson’s correlation coefficient (r) to investigate the pairwise correlations between the cytokine responses selected to be included in the risk prediction model. Correlations were visualized using a heatmap, with stronger correlations (r closer to 1) represented in shades closer to red and weaker correlations (r closer to 0) appearing closer to blue. We hoped to discern the intercorrelation patterns and potential clustering among the cytokine responses.

We aimed to construct prediction models using the selected cytokine responses to predict the risk of having SARS-CoV-2 infection severity of severe/death as opposed to mild/moderate. We did not incorporate other variables such as age, sex, or comorbidities/CCI into the prediction models because our objective was to assess cytokines as independent predictors of SARS-CoV-2 severity. Additionally, we also hoped to identify which variables contribute the most to the prediction of the severity of SARS-CoV-2 infection. However, given the intercorrelated nature of the cytokine responses, building prediction models using all the selected cytokine responses may lead to overfitting problems. Therefore, we decided to employ a penalized regression method, the least absolute shrinkage and selection operator (LASSO) logistic regression, to perform variable selection and regularization to achieve better model performance [12].

To construct the LASSO model, we logarithmically transformed the cytokine concentrations. The optimal penalty parameter was determined via tenfold cross-validation and then used to construct the LASSO model in the training set [13]. The discriminative ability of the LASSO model in the test set was evaluated using the ROC and AUC [14]. We assessed the calibration of the model using the Hosmer–Lemeshow tests. LASSO regression was conducted using the GLMNET package in R. Cytokine responses chosen through LASSO regression were presented with the absolute coefficient value for each variable indicated.

To offer an alternative and complementary analysis to the LASSO regression, we also leveraged a random forest model [15, 16] and the eXtreme Gradient Boosting (XGBoost) [17] using the selected cytokine responses to predict the severity of SARS-CoV-2 infection. The same training and test sets were used. We ranked the importance of the selected cytokine responses based on their ability to discriminate between patients having severe/death outcome as opposed to mild/moderate outcome. In the random forest model, feature importance was determined by the mean decrease in Gini [15], while in the XGBoost model, it was determined by the mean absolute SHapley Additive exPlanations (SHAP) value [18]. Similarly, we used ROC and AUC to evaluate the discriminative ability of the random forest model and XGBoost model in the test set and presented the most significant cytokine predictors and their feature importance in a table. We also assessed the calibration of the models using the Hosmer–Lemeshow tests.

Finally, we visualized the feature importance of each cytokine in the Lasso, random forest, and XGBoost models to assess the consistency of variable importance between the three methods and examine whether there is evidence that these cytokines were genuine predictors or selected by chance.

## Results

### Descriptive analysis

The baseline characteristics of the study subjects stratified by the severity of SARS-CoV-2 Infection are presented in Table 1. Age distributions were overall comparable between the mild/moderate and severe/death groups. However, the severe/death group had a higher proportion of participants aged 80 and above (16.7%) and fewer below 40 years (2.4%) than the mild/moderate group (aged 80 and above: 5.6%, below 40 years: 8.8%). A greater percentage of participants in the severe/death group had a CCI of 3 or higher (69.1%), indicating higher disease morbidity at the time of SARS-CoV-2 infection, compared to the mild/moderate group (48.7%). A higher proportion of the severe/death group was inpatients (97.6%) compared to the mild/moderate group (82.5%). The distribution of sexes was consistent across both groups.

**Table 1:** Baseline characteristics of study population stratified by the severity of SARS-CoV-2 infection

Variables	Mild/moderate (*n* = 160)	Severe/death (*n* = 42)
**Age**		
< 30	2 (1.3%)	0 (0.0%)
30 and < 40	12 (7.5%)	1 (2.4%)
40 and < 50	8 (5.0%)	3 (7.1%)
50 and < 60	28 (17.5%)	6 (14.3%)
60 and < 70	50 (31.3%)	11 (26.2%)
70 and < 80	51 (31.9%)	14 (33.3%)
> = 80	9 (5.6%)	7 (16.7%)
**CCI**		
0	35 (21.9%)	6 (14.3%)
1–2	47 (29.4%)	7 (16.7%)
3–4	37 (23.1%)	13 (31.0%)
5 +	41 (25.6%)	16 (38.1%)
**Race/Ethnicity**		
Hispanic	7 (4.4%)	4 (9.5%)
Non-Hispanic black	66 (41.3%)	15 (35.7%)
Non-Hispanic white	74 (46.3%)	19 (45.2%)
Other	13 (8.1%)	4 (9.5%)
**Cohort**		
In-Patient	132 (82.5%)	41 (97.6%)
Out-Patient	28 (17.5%)	1 (2.4%)
**Sex**		
Female	15 (9.4%)	3 (7.1%)
Male	145 (90.6%)	39 (92.9%)

Data are presented as n (%)

The baseline cytokine responses of the study subjects stratified by the severity of SARS-CoV-2 Infection are presented in Table 2. Several cytokines have more than 40% of their values out of detection limits, including FGF-2, IL-1β, IL-4, IL-5, IL-6, IL-9, IL-12p70, IL-21, IL-22, IL-23, IL-27, IL-31, NGF-β, TNF-β, and VEGF-D. Overall, these data suggest that individuals with different illness severity display distinct cytokine expression patterns at the time of presentation.

**Table 2:** Cytokine levels of study population stratified by the severity of SARS-CoV-2 infection

Cytokines	Mild/moderate (*n* = 160)	Severe/death (*n* = 42)
BDNF	73.02 (34.59, 187.07)	110.56 (46.60, 301.73)
EGF	21.67 (6.54, 66.32)	18.69 (5.53, 37.66)
Eotaxin	78.24 (36.08, 141.35)	94.86 (55.38, 163.57)
FGF-2	OOR < (OOR <, 11.38)	OOR < (OOR <, 6.80)
GM-CSF	43.85 (7.08, 220.38)	21.66 (OOR <, 67.10)
GRO-α	7.50 (OOR <, 27.42)	7.63 (OOR <, 21.15)
HGF	272.18 (120.89, 698.81)	609.71 (243.60, 1092.81)
IFN-α	1.17 (OOR <, 6.05)	0.85 (OOR <, 3.32)
IFN-γ	16.66 (4.67, 57.78)	18.90 (8.86, 41.84)
IL-1-α	0.41 (OOR <, 2.13)	0.36 (OOR <, 1.86)
IL-1-β	5.80 (OOR <, 25.64)	3.65 (OOR <, 13.68)
IL-1RA	1078.73 (498.40, 2100.30)	1704.74 (796.54, 3459.01)
IL-2	14.39 (OOR <, 62.00)	6.24 (OOR <, 16.11)
IL-4	OOR < (OOR <, 42.16)	OOR < (OOR <, OOR <)
IL-5	1.35 (OOR <, 35.47)	OOR < (OOR <, 13.73)
IL-6	25.28 (OOR <, 219.44)	26.22 (OOR <, 80.60)
IL-7	3.62 (1.36, 9.35)	3.09 (1.54, 6.60)
IL-8	4.79 (OOR <, 13.35)	6.75 (2.47, 14.50)
IL-9	OOR < (OOR <, 17.40)	OOR < (OOR <, OOR <)
IL-10	1.79 (OOR <, 7.34)	2.39 (0.93, 4.96)
IL-12p70	0.68 (OOR <, 2.91)	0.41 (OOR <, 1.12)
IL-13	8.73 (OOR <, 29.76)	6.32 (2.42, 16.44)
IL-15	20.53 (1.88, 116.78)	9.33 (1.10, 31.35)
IL-17A	17.11 (1.27, 78.41)	6.20 (OOR <, 27.34)
IL-18	33.40 (16.47, 60.64)	36.83 (24.05, 67.07)
IL-21	OOR < (OOR <, 94.50)	OOR < (OOR <, 17.04)
IL-22	OOR < (OOR <, 44.69)	OOR < (OOR <, 12.42)
IL-23	OOR < (OOR <, 88.47)	OOR < (OOR <, OOR <)
IL-27	33.31 (OOR <, 285.85)	OOR < (OOR <, 65.76)
IL-31	OOR < (OOR <, 126.79)	OOR < (OOR <, OOR <)
IP-10	124.08 (55.83, 596.16)	660.36 (212.48, 1617.52)
LIF	10.06 (3.83, 46.29)	7.56 (4.28, 19.98)
MCP-1	97.56 (51.85, 188.78)	183.26 (84.34, 293.89)
MIP-1-α	14.38 (3.89, 42.40)	12.57 (8.81, 38.50)
MIP-1-β	78.72 (31.46, 132.79)	92.78 (66.13, 144.45)
NGF-β	6.19 (OOR <, 25.01)	OOR < (OOR <, 6.19)
PDGF-BB	234.62 (95.45, 602.95)	267.38 (110.06, 1239.12)
PIGF-1	13.62 (OOR <, 41.78)	19.27 (OOR <, 57.38)
RANTES	612.82 (339.06, OOR >)	696.85 (398.89, OOR >)
SCF	15.10 (7.20, 31.94)	15.15 (9.25, 31.35)
SDF-1-α	675.14 (331.74, 978.67)	794.22 (526.88, 1263.75)
TNF-α	5.84 (0.86, 31.01)	4.72 (0.81, 9.44)
TNF-β	OOR < (OOR <, OOR <)	OOR < (OOR <, OOR <)
VEGF-A	184.47 (86.27, 490.23)	325.19 (194.84, 790.06)
VEGF-D	OOR < (OOR <, 40.67)	7.62 (OOR <, 35.50)

Data are presented as median (IQR)

OOR <, below the lower detection limit; OOR >, above the upper detection limit

The training and test sets were comparable in terms of SARS-CoV-2 severity and baseline characteristics, as evidenced by similar distributions across groups (Supplementary Table 1).

Using these data, we identified 10 cytokines that differentiated illness severity in a univariate analysis (Table 3). We selected cytokines with AUC 95% CI lower bound > 0.5, which indicates significant predictive power over chance. IP-10 has the highest AUC (0.707, 95% CI: 0.622–0.791), followed by HGF (0.649, 95% CI: 0.554–0.744) and MCP-1 (0.635, 95% CI: 0.534–0.736). The 10 cytokines in Table 3 were selected to be included in the risk prediction models. These data suggest that certain cytokines have significant predictive power in distinguishing between different severities of SARS-CoV-2 infection. In comparison to these cytokines, using CCI alone achieved an AUC of 0.603 (95% CI: 0.511–0.696), which is less than or at most comparable to the predictive performance of individual cytokines such as IP-10, HGF, and MCP-1.

**Table 3:** ROC curve analysis by cytokines to predict the severity of SARS-CoV-2 infection

Cytokines	Cutoff Value	AUC (95% CI)	Sensitivity	Specificity	Youden Index
GM-CSF	26.805	0.599 (0.507, 0.691)	0.595	0.619	0.214
HGF	235.305	0.649 (0.554, 0.744)	0.810	0.463	0.272
IL-1RA	1767.135	0.599 (0.504, 0.695)	0.500	0.706	0.206
IL-2	10.950	0.609 (0.516, 0.702)	0.667	0.575	0.242
IL-15	14.000	0.597 (0.505, 0.689)	0.643	0.588	0.230
IL-17A	12.305	0.601 (0.507, 0.695)	0.619	0.575	0.194
IL-27	71.405	0.593 (0.511, 0.675)	0.810	0.456	0.266
IP-10	200.975	0.707 (0.622, 0.791)	0.762	0.619	0.381
MCP-1	156.295	0.635 (0.534, 0.736)	0.595	0.694	0.289
VEGF-A	180.780	0.621 (0.529, 0.713)	0.786	0.500	0.286

**Fig. 1:**
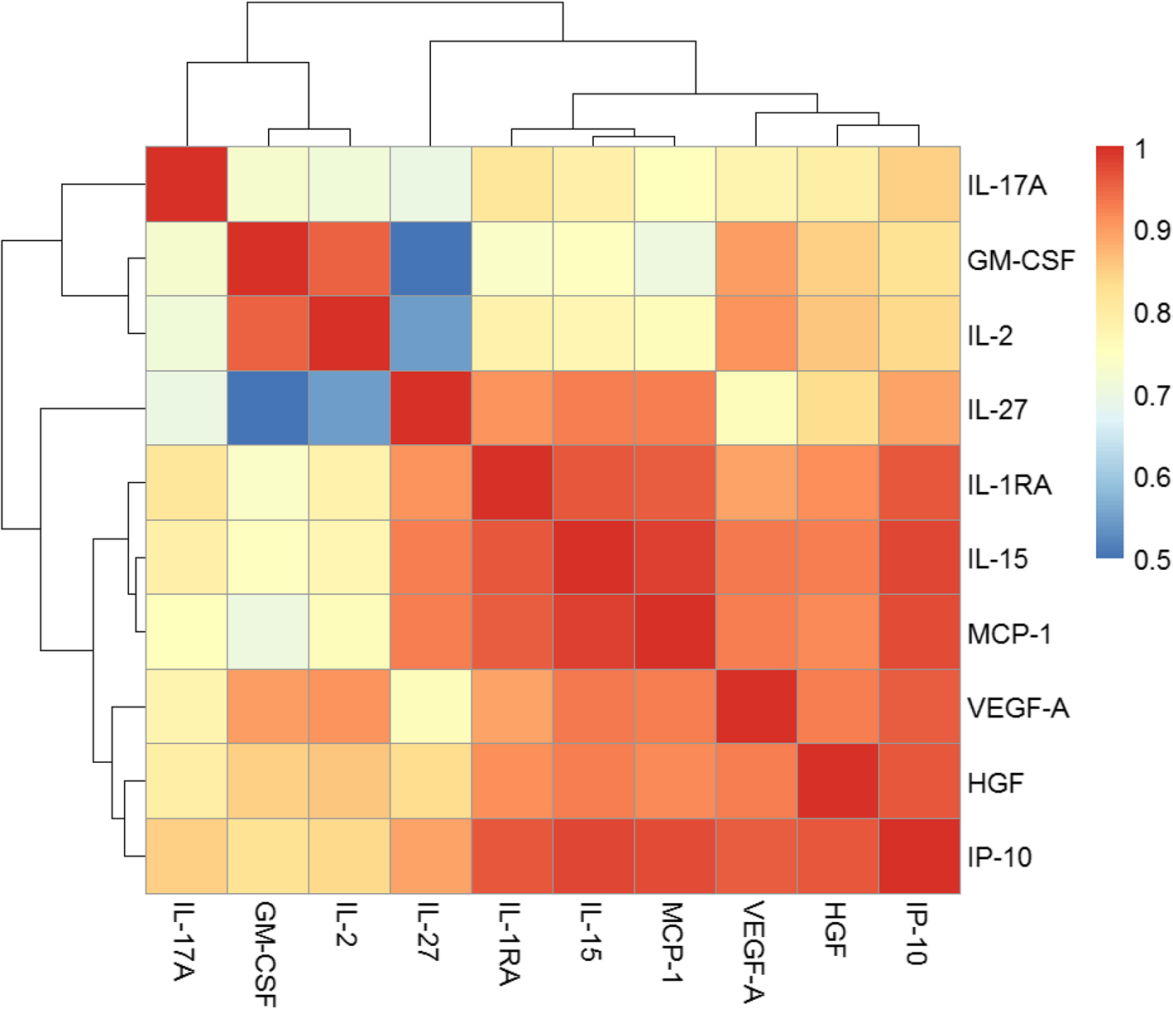
Heatmap of correlations between 10 cytokines with individual AUC 95% CI lower bounds exceeding 0.5

We also compared the correlation between selected cytokines of interest from the univariate analysis (Fig. 1). We identified two clusters of intercorrelated cytokines, as indicated by the two predominant red blocks in the heatmap. In the larger red block, IL-27, IL-1RA, IL-15, MCP-1, VEGF-A, HGF, and IP-10 exhibited strong correlations with each other. Meanwhile, in the smaller red block, IL-2 and GM-CSF were strongly correlated.

### LASSO regression

**Fig. 2:**
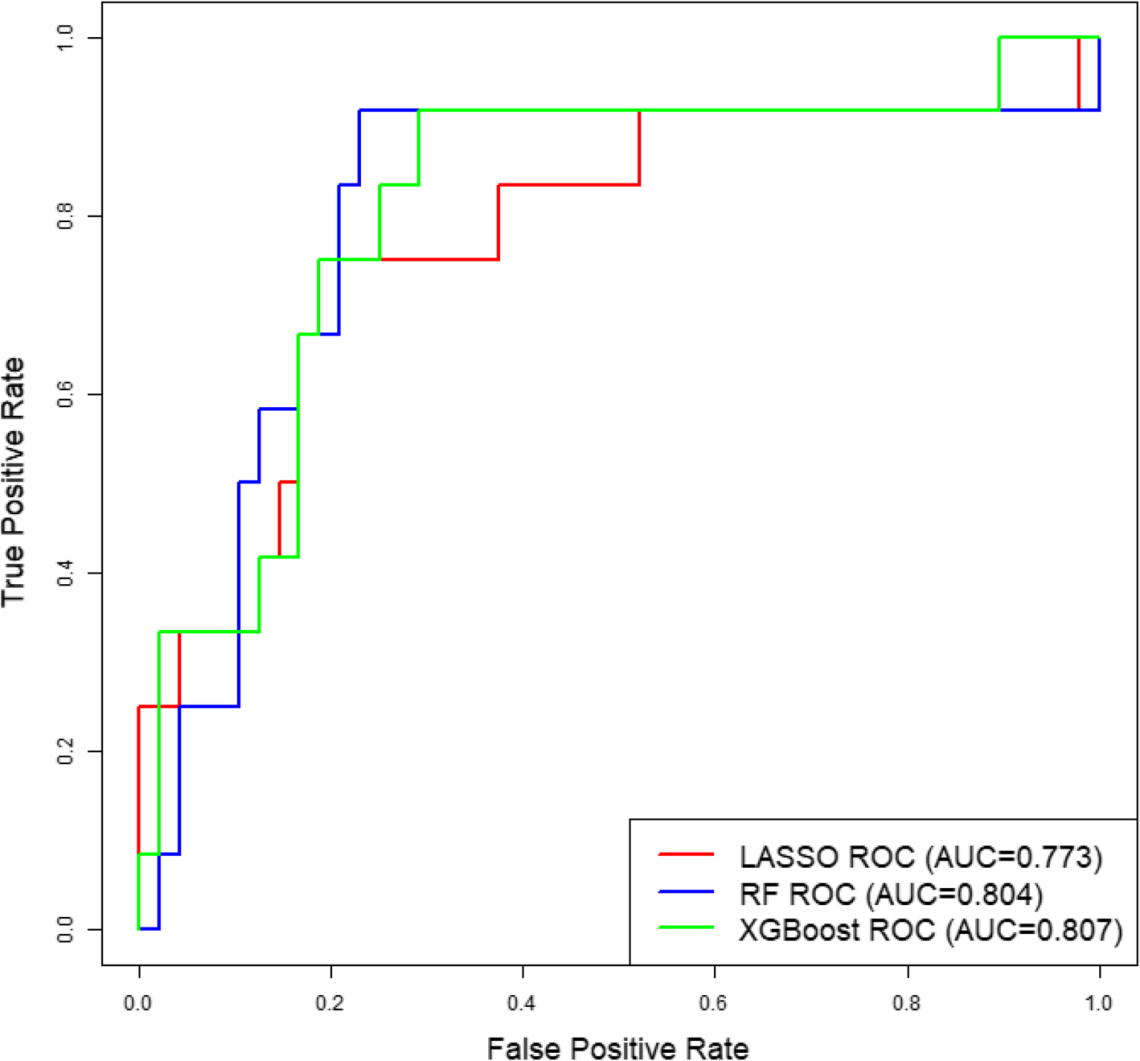
ROC curves for the LASSO, RF, and XGBoost models

**Fig. 3:**
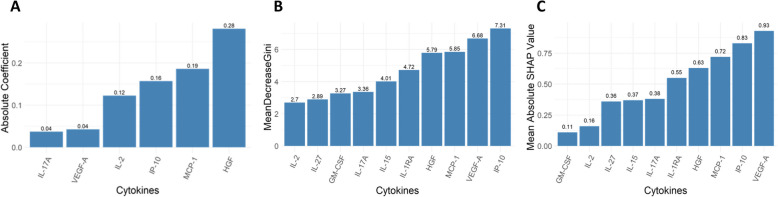
Feature importance of each cytokine in the risk prediction models. **A** LASSO model. **B** RF model. **C** XGBoost model

Using LASSO regression, we identified a set of cytokines effective for outcome prediction. Six cytokines were selected by the LASSO model, based on their absolute regression coefficient values, with the strongest predictive power provided by HGF (0.28), followed by MCP-1 (0.19), IP-10 (0.16), IL-2 (0.12), VEGF-A (0.04), and IL-17A (0.04) (Fig. 3a). These cytokines predict outcomes in this initial model with an AUC for the test set of 0.773 (Fig. 2, 95%CI: 0.640–0.967). The Hosmer–Lemeshow test p-value was 0.263 (Table 4).

**Table 4:** ROC curve analysis and Hosmer–Lemeshow *p*-value by models to predict the severity of SARS-CoV-2 infection

Models	AUC (95% CI)	Sensitivity	Specificity	Youden Index	Hosmer–Lemeshow *p*-value
LASSO	0.773 (0.601, 0.944)	0.750	0.792	0.542	0.263
RF	0.804 (0.640, 0.967)	0.917	0.771	0.688	0.155
XGBoost	0.807 (0.656, 0.959)	0.917	0.708	0.625	0.218

### Random forest model and XGBoost model

To further bolster our statistical conclusions, we performed random forest and XGBoost modeling to predict outcomes using the same data. The random forest model identified a set of cytokines with similar predicted test effectiveness as in LASSO regression (Fig. 2, AUC 0.804, 95%CI: 0.601–0.944). The feature importance of each cytokine is presented as the mean decrease in Gini (Fig. 3b). Likewise, XGBoost identified a cytokine set with similar performance (Fig. 2, AUC 0.807, 95%CI: 0.656–0.959). Here, the feature importance of each cytokine is presented as mean absolute SHAP value (Fig. 3c). In the random forest model, IP-10 has the highest feature importance, followed by VEGF-A, MCP-1, HGF, IL-1-RA, IL-15, IL-17A, GM-CSF, IL-27, and IL-2. In the XGBoost model, VEGF-A has the highest feature importance, followed by IP-10, MCP-1, HGF, IL-1-RA, IL-17A, IL-15, IL-27, IL-2, and GM-CSF. The Hosmer–Lemeshow test p-values for the random forest and XGBoost models were 0.155 and 0.218, respectively (Table 4). The AUC, sensitivity, specificity, Youden index, and Hosmer–Lemeshow test p-values for the LASSO, RF, and XGBoost models are detailed in Table 4.

## Discussion

In this study, we identified a set of peripheral blood cytokines that effectively predict the severity of SARS-CoV-2 infection in a Veteran population. The AUC for the LASSO model is greater than 0.7, which is considered acceptable, while the AUC values for the random forest and XGBoost models exceed 0.8, indicating excellent discriminative ability. In addition, the models also demonstrate good calibration, as reflected by their Hosmer–Lemeshow p-values being greater than 0.05. The consistency of the feature importance of each cytokine in the three models is particularly noteworthy. Specifically, cytokines such as Interferon gamma-induced protein 10 (IP-10), Vascular Endothelial Growth Factor A (VEGF-A), Monocyte Chemoattractant Protein 1 (MCP-1), and Hepatocyte Growth Factor (HGF) were repeatedly highlighted as important predictors, suggesting their robust role in the predictive models. This consistency across multiple models suggests that cytokine profiles may predict the severity of SARS-CoV-2 infection after validation in larger and more diverse cohorts.

The peripheral blood cytokines identified to be key risk predictors for the severity of SARS-CoV-2 infection in our study align with observations from several studies that also identified many of the same cytokines. Yang et al. reported that plasma IP-10 and MCP-3 levels are highly associated with illness severity and predict the progression of COVID-19 [6]. Similarly, Chen et al. identified IP-10 and MCP-1 as key biomarkers for COVID-19 severity [19]. Furthermore, Perreau et al. demonstrated that the cytokines HGF and CXCL13 are predictive of both the severity and mortality in COVID-19 patients [20]. Understanding these immune patterns may provide improved clinical decision-making at the individual level and improved resource allocation at a population level. Of the 10 cytokines identified as individual predictors, several (IP-10, IL-2, IL-15, IL-17A) are dependent on effective adaptive T cell responses to reduce inflammation and severity of disease. These echo multiple studies, including predictive models [21], observations that T cell memory responses to SARS-CoV-2 reduce the severity of disease in convalescent individuals [22] and after vaccination across serotypes [23, 24]. These data provide further evidence of the key importance of SARS-CoV-2 vaccination in preventing mortality from SARS-CoV-2 in our US Veteran population.

This study has several strengths. First, we utilized a comprehensive panel of 45 cytokines, allowing for a thorough investigation of potential biomarkers. This broad approach increases the likelihood of identifying key cytokines for predicting the risk of severe SARS-CoV-2 infection. Second, our study employed a robust methodological framework, utilizing LASSO logistic regression, random forest, and XGBoost models to analyze a complex dataset with multiple predictors. This multi-method approach not only provided a comprehensive analysis but also served to cross-validate our findings, enhancing their reliability. The discriminative ability observed in the test set (77.3%, 80.4%, and 80.7%, respectively) is indicative of the effectiveness of our models. In addition, the study draws data from 15 different VA medical centers across the United States, providing a geographically and racially diverse sample. Enrollment took place from June 2020 to September 2022, and so participants were exposed to a wide range of SARS-CoV-2 variants. Moreover, the VASIC is a validated tool specifically tailored to assess COVID-19 severity, which reduces misclassification and enhances the accuracy of severity assessments. Finally, we had comprehensive data from the medical records on participant characteristics and outcomes, and the multi-point follow-up in the EPIC^3^ study enables a detailed capture of disease progression over time.

While the study offers valuable insights, certain limitations must be acknowledged. Firstly, the focus on the US Veteran population, while providing detailed insights for this demographic, might limit the generalizability of our findings to the wider population. The diverse health profiles and experiences of Veterans may not fully represent the broader spectrum of patients affected by COVID-19, especially female populations. Additionally, our study population also differs in terms of baseline characteristics from the participants of the broader EPIC^3^ study. Among those who participated in EPIC^3^ as inpatient or outpatient participants and had a positive RT-PCR test at baseline, only 25.2% were inpatients, whereas in our study population, where baseline multiplex cytokine measurements were available, 85.6% were inpatients. This discrepancy arises because inpatients are more likely to undergo comprehensive biomarker assessments. In the future, expanding cytokine measurements to a larger and more diverse participant pool will be essential for enhancing the generalizability of our findings. Moreover, the simplification of the Veterans Affairs Severity Index for COVID-19 into a binary outcome may have obscured more subtle gradations in illness severity. This could impact the applicability of our findings to clinical scenarios where such nuances are critical. Another limitation is the dependency on the Luminex assay platform for cytokine measurement, which could pose challenges in replicating our results in settings where different technologies or assays are used. Furthermore, this study only leverages baseline cytokine measurements, and we are not capturing the dynamic changes in cytokine levels that occur over time or at the time of symptom onset, which may affect the performance of risk prediction models.

To address these limitations and build on our findings, future research should focus on a more diverse and representative sample of the population, which would enhance the external validity and applicability of the results. Longitudinal studies would provide valuable insights into the temporal dynamics of cytokine profiles and their correlation with disease progression and recovery. Additionally, incorporating other biomarkers and clinical parameters into the analysis could offer a more comprehensive understanding of COVID-19 and its myriad presentations. Such integrative studies could further refine the predictive models and potentially uncover new therapeutic targets or diagnostic markers.

In conclusion, our study demonstrates that peripheral blood cytokine profiles are effective predictors of SARS-CoV-2 infection severity among US Veterans. Using multiple methods, we identified key cytokines correlated with severe outcomes. Future research should focus on validating these results in larger cohorts and exploring the underlying mechanisms of these cytokines in COVID-19 progression, paving the way for targeted treatment approaches.

## Supplementary Information

Supplementary Material 1

## Data Availability

Participating Veterans contributed data by completing questionnaires, either conducted as interviews or filled out by the Veterans themselves, and by providing biospecimens; clinical data assessment was conducted accessing comprehensive VHA Electronic Health Records (EHR).
