# Supplementary material for: Peripheral blood cytokine profiles predict the severity of SARS-CoV-2 infection: an EPIC3 study analysis
Source: BMC Infect Dis. 2025 May 8;25:677. doi: 10.1186/s12879-025-10914-6 (PMC12063214; doi:10.1186/s12879-025-10914-6)
Supplement: Supplementary file 1 — Supplementary Material 1 [file 12879_2025_10914_MOESM1_ESM.docx]

**Appendix**

Supplementary Table 1: Severity of SARS-CoV-2 Infection and Baseline Characteristics of Study Population in the Training and Test Sets.

| **Variables** | **Training set (n=142)** | **Test set (n=60)** |
| --- | --- | --- |
| **Age** |  |  |
| <30 | 1 (0.7%) | 1 (1.7%) |
| 30 and <40 | 9 (6.3%) | 4 (6.7%) |
| 40 and <50 | 8 (5.6%) | 3 (5.0%) |
| 50 and <60 | 24 (16.9%) | 10 (16.7%) |
| 60 and <70 | 43 (30.3%) | 18 (30.0%) |
| 70 and <80 | 46 (32.4%) | 19 (31.7%) |
| >=80 | 11 (7.7%) | 5 (8.3%) |
| **CCI** |  |  |
| 0 | 29 (20.4%) | 12 (20.0%) |
| 1-2 | 39 (27.5%) | 15 (25.0%) |
| 3-4 | 33 (23.2%) | 17 (28.3%) |
| 5+ | 41 (28.9%) | 16 (26.7%) |
| **Race/Ethnicity** |  |  |
| Hispanic | 7 (4.9%) | 4 (6.7%) |
| Non-Hispanic black | 57 (40.1%) | 24 (40.0%) |
| Non-Hispanic white | 65 (45.8%) | 28 (46.7%) |
| Other | 13 (9.2%) | 4 (6.7%) |
| **Cohort** |  |  |
| In-Patient | 121 (85.2%) | 52 (86.7%) |
| Out-Patient | 21 (14.8%) | 8 (13.3%) |
| **Sex** |  |  |
| Female | 13 (9.2%) | 5 (8.3%) |
| Male | 129 (90.8%) | 55 (91.7%) |
| **Severity of SARS-CoV-2 Infection** |  |  |
| Mild/moderate | 114 (80.3%) | 46 (76.7%) |
| Severe/death | 28 (19.7%) | 14 (23.3%) |

Data are presented as n (%).
